# Benchmarking total hip replacement constructs using noninferiority analysis: the New Zealand joint registry study

**DOI:** 10.1186/s12891-021-04602-0

**Published:** 2021-08-21

**Authors:** Michael Wyatt, Chris Frampton, Michael Whitehouse, Kevin Deere, Adrian Sayers, David Kieser

**Affiliations:** Massey University, Manawatu Campus, Palmerston North, New Zealand

**Keywords:** Total hip replacement, Noninferiority analysis, Benchmarking

## Abstract

**Background:**

The aim of this study was to compare the relative performance of total hip replacement constructs and discern if there is substantial variability in performance in currently commonly used prostheses in the New Zealand Joint Registry (NZJR) using a noninferiority analysis.

**Methods:**

All patients who underwent a primary total hip replacement (THR) registered in the NZJR between 1st January 1999 to June 2020 were identified. Using a noninferiority analysis, the performance of hip prostheses were compared with the best performing contemporary construct. Construct failure was estimated using the 1-Kaplan Meier survival function method to estimate net failure. The difference in failure between the contemporary benchmark and other constructs was examined.

**Results:**

In total 135,432 THR were recorded comprising 1035 different THR constructs. Notably 328 constructs were used just once. Forty-eight constructs (62,251 THR) had > 500 procedures at risk at 3 years post-primary of which 28 were inferior by at least 20% relative risk of which, 10 were inferior by at least 100% relative risk. Sixteen constructs were identified with > 500 procedures at risk at 10 years with 9 inferior by at least 20%, of which one was inferior by > 100% relative risk. There were fewer constructs noninferior to the best practice benchmark when we performed analysis by gender. In females at 10 years, from 5 constructs with > 500 constructs at risk, 2 were inferior at the 20% margin. In males at 10 years, there were only 2 eligible constructs of which one was inferior at the 20% margin.

**Conclusions:**

We discerned that there is substantial variability in construct performance and at most time points, just over half of constructs are inferior to the best performing construct by at least 20%. These results can facilitate informed decision-making when considering THR surgery.

**Supplementary Information:**

The online version contains supplementary material available at 10.1186/s12891-021-04602-0.

## Background

Not all total hip replacements perform equally well and there have been some high profile failures which have included the 3M™ Capital™ cemented hip implant (3 M Healthcare Ltd., Minnesota, USA) and metal-on-metal bearings [1–4]. Within contemporary and commonly used hip replacements there is substantial variation in performance [5]. How apparent this is to patients, clinicians and health funders is unknown. This lack of transparency is a profoundly important issue with respects to medical device safety as recently highlighted in the Cumberlege review [6].

The National Joint Registry of New Zealand (NZJR) monitors the performance of joint replacement implants and identifies poorly performing implants. It has not as yet focused on identifying exceptionally-performing implants or comparing implants to the best performing in a time or gender specific strata. The NZJR publishes the unadjusted failure rates expressed as a prosthesis time incidence rate (PTIR) of the THR brand combinations used in its annual reports. In New Zealand the Pharmaceutical Management Agency (Pharmac) is the agency that decides which THR products are subsidized for use in public hospitals (pharmacy.govt.nz; www.pharmac.govt.nz/devices-forum-summary-auckland.pdf). Understanding which devices should receive subsidies is important to ensure joint replacement is as cost effective as possible. In the private sector the decision of which THR implants are used is at the surgeon’s discretion.

Promoting good practice and implant choices has been done by organizations such as the Orthopaedic Data Evaluation Panel (ODEP) in the UK [7], in Australia, the Australian superior clinical performance programme [8] but these commonly use an arbitrarily defined static benchmark.

Without randomized controlled trial evidence, prospective national registers of joint replacement (national joint registries) provide the best current evidence base of THR construct performance. Like all observational data it has inherent limitations that potentially affect the interpretation of the outcomes of prosthesis or prosthesis constructs. The PTIRs reported by the NZJR gives an indication of performance in absolute terms but not a direct “head-to head” comparison. Sayers et al. proposed an analysis paradigm which used a noninferiority design against an external benchmark [9]. In a noninferiority trial with failure as an outcome, a comparator and reference can be compared to ensure that the comparator treatment is within a clinically acceptable range (noninferiority margin) of performance [10]. A recent study using the National Joint Registry applied the principles of a noninferiority trial to a best practice dynamic benchmarking setting [5]. The choice of the appropriate contemporary reference to define the best practice benchmark was the construct, used in sufficient numbers to protect against chance, clustering and surgical performance variation, with the lowest failure rate. Reports were stratified by age and gender as these are known to influence prosthesis failure rates [11] and reported at a variety of time points for purposes of comparison and provision of information to stakeholders. There has recently been great variability demonstrated in THR construct performance in the UK at 3, 5, 7 and 10 year time points [5]. Whether similar findings occur in New Zealand is unknown.

The aim of this study therefore was to compare the performance of THR constructs to the best-performing constructs using a noninferiority analysis and illustrate variability in performance. Stem, bearing and cup brand combinations (constructs) were examined against noninferiority margins of 20 and 100% relative risk (i.e. double the revision rate) at 3, 5, 7 and 10 years following implantation.

## Methods

### Patients and data sources

The NZJR was established in 1998 and has a > 96% data capture rate of all joint replacement surgeries [12]. Prospective entry of data into the NZJR is a mandatory requirement of all members of the New Zealand Orthopaedic Association with all data secured in Christchurch, New Zealand. One of the authors (CF) accessed the database to acquire data specifically for this study. Deidentified data of all patients undergoing primary THR from the NZJR inception to 1st June 2020 was available for analysis and the NZJR is linked directly with the NZ database for births and deaths. We included metal on polyethylene (MP), ceramic on polyethylene (CP) and ceramic on ceramic (CC) bearing surfaces but excluded other hard-on-hard bearings such as metal-on-metal.

### Primary exposure

The primary exposure used was the THR construct defined by the femoral stem, acetabular cup and bearing surface combination. These were defined using data recorded by the NZJR and based on the catalogue numbers of individual THR components.

### Statistical methods

Construct failure was estimated using the 1-Kaplan-Meier method, that is, an estimate of net failure [13]. Failure was defined using the first linked surgical revision; patients were censored at death. A revision was defined as a new operation in a previous THR during which one or more of the components was exchanged, removed, manipulated or added. It included excision arthroplasty and amputation, but not soft tissue procedures.

The reference construct was that with the lowest failure rate with at least 1000 patients at risk at the time point of interest for all procedures and for each gender-specific stratum. The choice of 1000 procedures of the same construct was based on simulation work by Sayers et al. [9] as if there are 1000 procedures at risk this will give rise to a CI width of ~ 3% (±1·5%). The difference in stratum-specific failure probabilities compared with the reference were calculated at 3, 5, 7 and 10 years for all prosthesis (stem–cup–bearing) combinations that had 500 or more patients and also then stratified by gender. The difference and 95% CI of the difference between the comparator construct and the reference construct was estimated at the specified time points. The standard error (SE) of the difference was calculated by a pooled estimate of the Greenwood SE [14]. A Wald test was performed to compare the difference between the reference and the test prosthesis.

The noninferiority margins chosen were 20 and 100% relative risk. The former represents the usual threshold used in clinical trials and the latter represents a doubling in cumulative probability of failure. Results are shown graphically at each time point for all comparator prosthesis constructs with at least 500 at risk. This number was chosen based on Sayers et al. [9] as this would give rise to an individual CI width of ~ 5% (±2·5%). The failure difference for each construct compared to the benchmark and the number of constructs still at risk are shown. This methodology complements the number of procedures at risk used by ODEP when evaluating devices at 10 years (www.odep.org.uk).

**Fig. 1:**
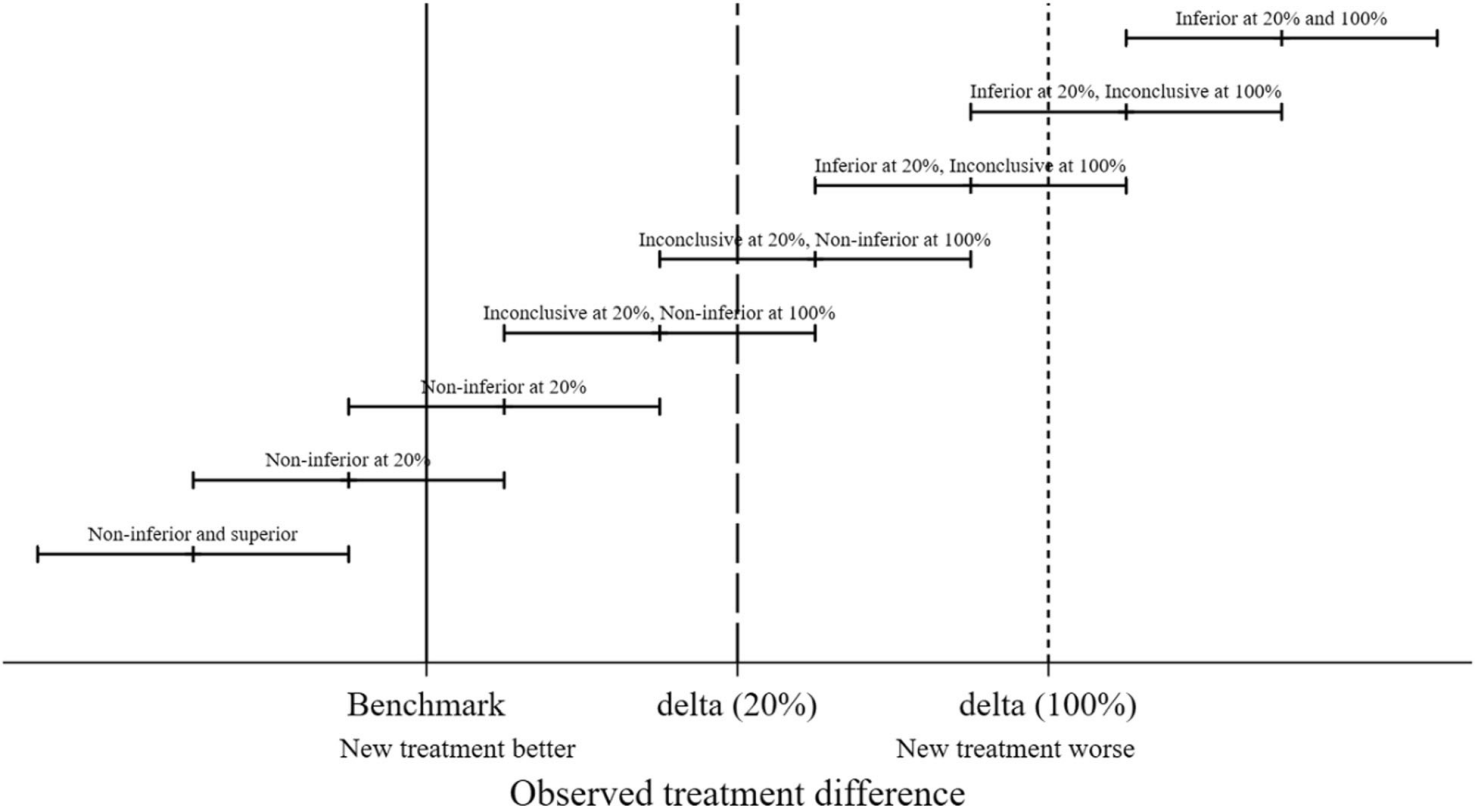
Schematic representation of inferiority and noninferiority

**Fig. 2:**
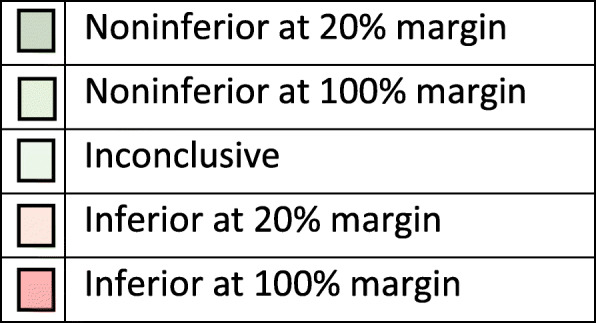
Key for colour coding in Figures 3-13. If no constructs were in a particular band the colour was not displayed

THR constructs were classified as noninferior, inconclusive or inferior by comparison with the two noninferiority margins and the classification shown in the five resultant groups. If the lower CI limit is above the 100% noninferiority margin, the construct was classified as inferior at the 100% margin. If the lower CI limit was above the 20% noninferiority margin but not above the 100% inferiority margin the construct was classified as inferior at the 20% margin. If the upper CI limit was below the 100% inferiority margin, the construct was defined as noninferior at the 100% margin. If the upper CI limit was below the 20% inferiority margin, the construct was defined as noninferior at the 20% margin. All the other results were determined to be inconclusive in terms of both 20 and 100% margins for noninferiority. Figure 1 provides a graphical representation of the rationale for the classification for a single noninferiority margin and Fig. 2 a key for subsequent figures.

## Results

There were 135,432 primary THR included in the NZJR from inception to 1st June 2020 of which 62,251 were available for the final analysis. In total 1035 different constructs were used at least once. Three hundred twenty-eight constructs were used just once. A detailed description of noninferiority across all procedures is provided. Constructs are described using the brand of stem and cup combination and the bearing surface couple. Bearings are combinations of either ceramic (C), metal (M), or polyethylene (P). Figures were also produced for stratification by gender.

### Noninferiority: all procedures

**Fig. 3:**
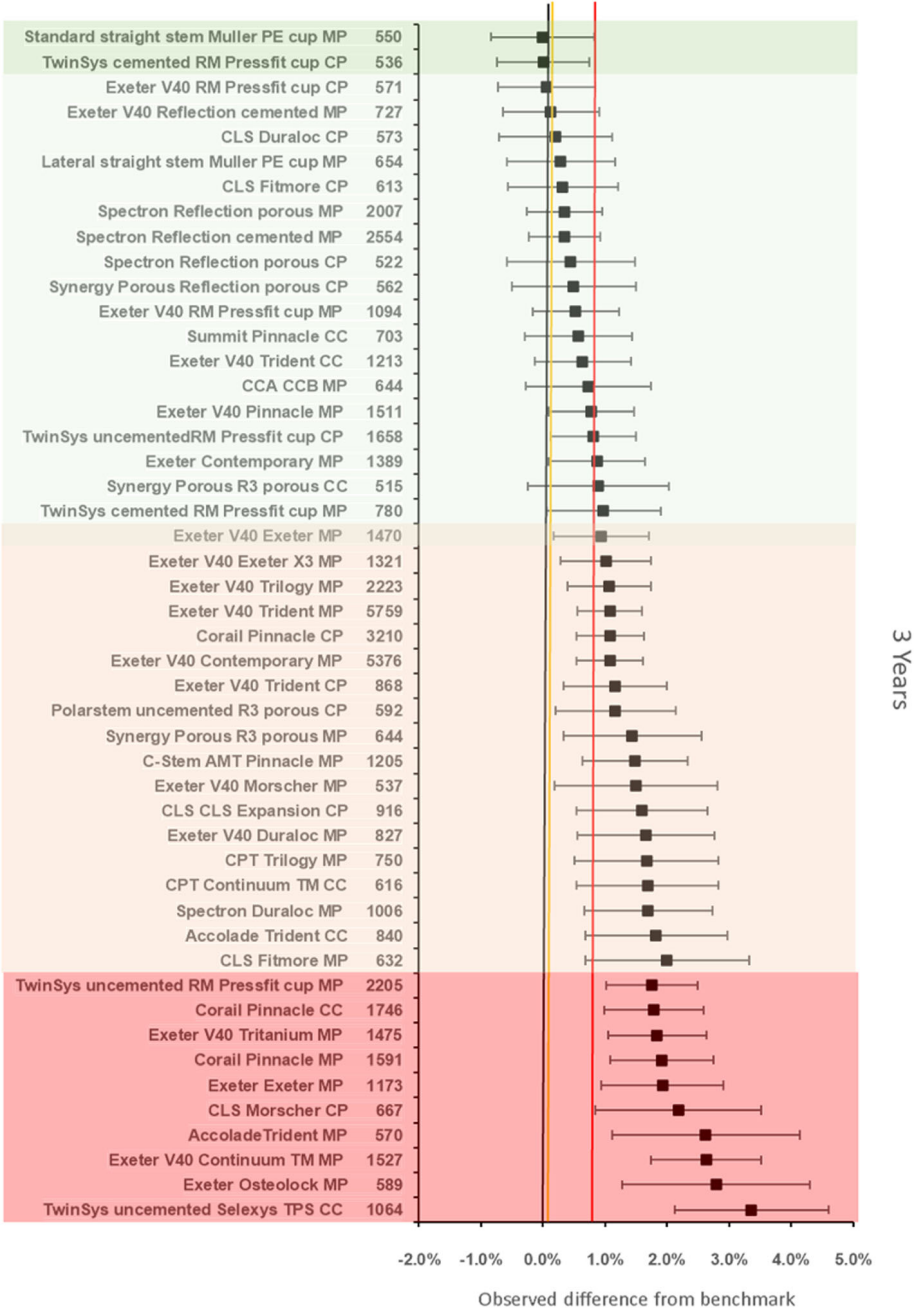
Difference in failure of implanted constructs compared with a contemporary reference (**MS30/Fitmore MP** (0.83, 95% CI 0.42–1.24)) at 3 years, using all stem-cup combinations with > 500 procedures remaining at risk. CC, ceramic-on-ceramic; CP, ceramic-on-polyethylene; MP, metal-on-polyethylene

The benchmark prosthesis construct at 3 years was identified as the MS30/Fitmore (Zimmer Ltd., Winterhur, Switzerland) metal on polyethylene. There were 1476 remaining at risk and the failure rate was 0·83% (95%CI 0.42–1.24). There were 48 other constructs with > 500 at risk. Twenty-eight were inferior by at least 20% relative risk of which 10 constructs were identified as inferior to the reference by > 100% relative risk. Two constructs were noninferior to the 100% margin (Fig. 3).

**Fig. 4:**
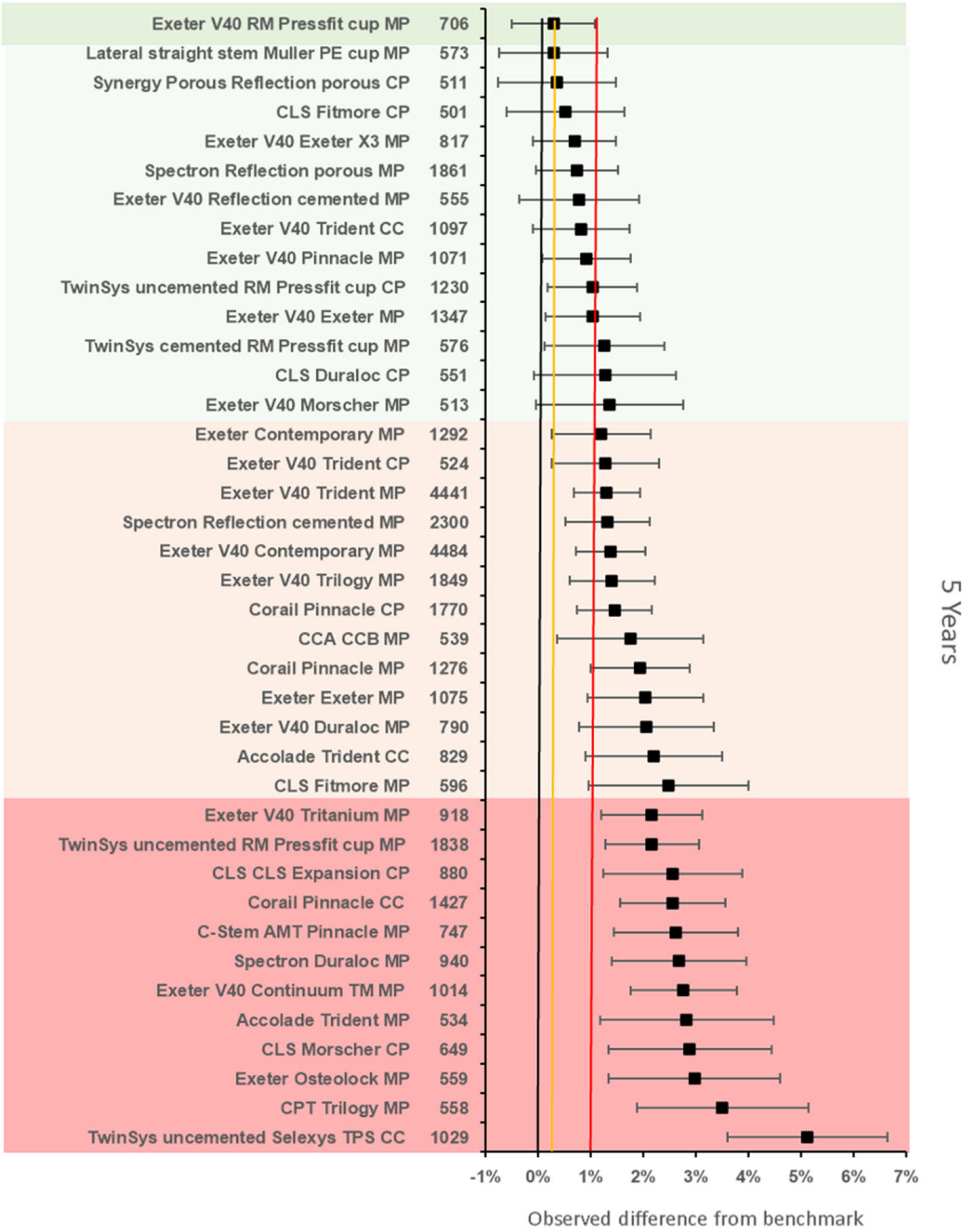
Difference in failure of implanted constructs compared with a contemporary reference (**MS30/Fitmore MP** (1.16, 95% CI 0.64–1.67)) at 5 years, using all stem-cup combinations with > 500 procedures remaining at risk. CC, ceramic-on-ceramic; CP, ceramic-on-polyethylene; MP, metal-on-polyethylene

The benchmark prosthesis construct at 5 years was again identified as the MS30/Fitmore metal on polyethylene. There were 1112 remaining at risk and the failure rate was 1·16% (95% CI 95% CI 0.64–1.67). There were 39 other constructs with > 500 at risk. Twenty-five constructs were inferior by at least 20% relative risk of which 12 constructs were inferior to the reference by > 100% relative risk. One construct was noninferior to the 100% margin (Fig. 4).

**Fig. 5:**
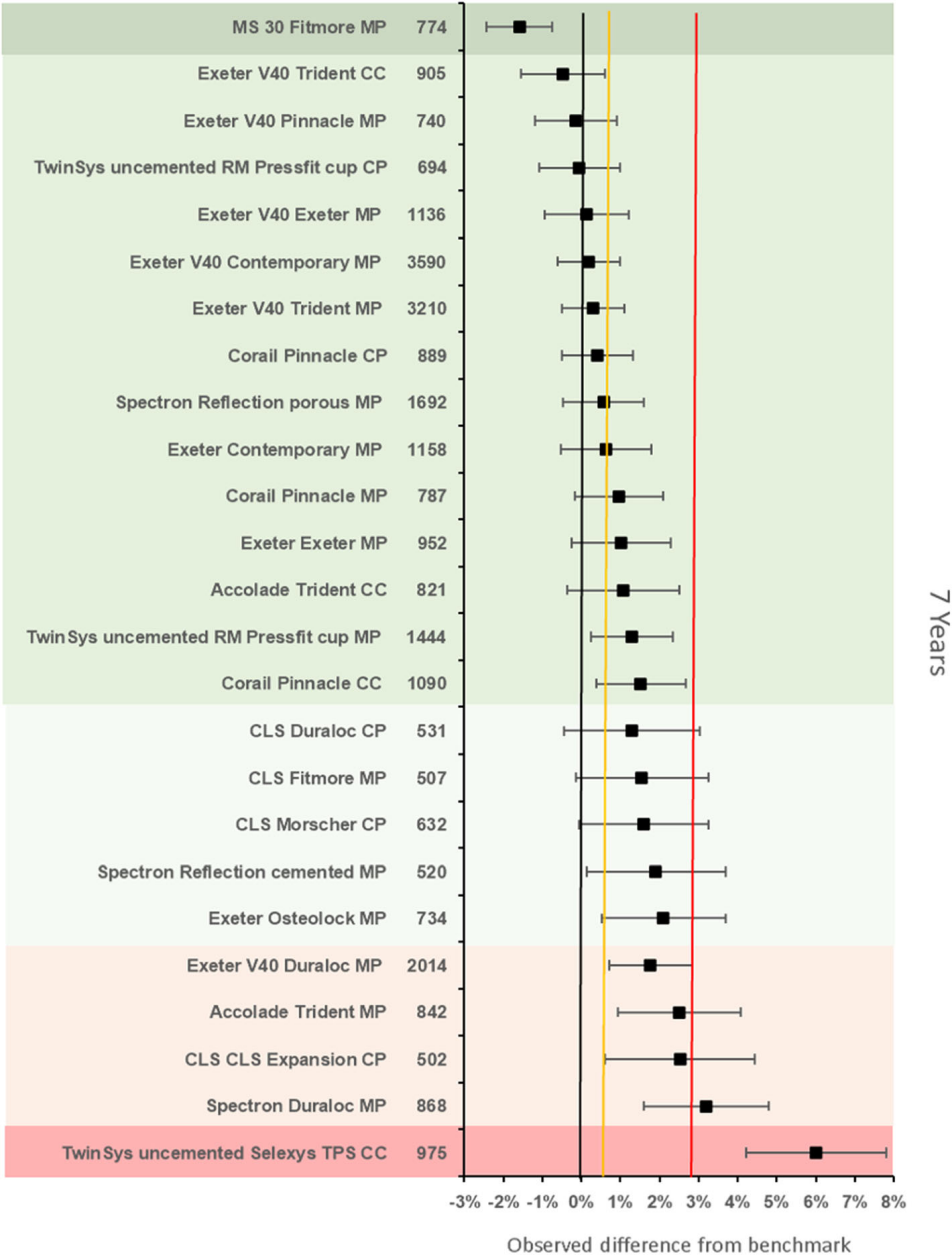
Difference in failure of implanted constructs compared with a contemporary reference (**ExeterV40/Trilogy MP** (2.74, 95% CI 2.08–3.40)) at 7 years, using all stem-cup combinations with > 500 procedures remaining at risk. CC, ceramic-on-ceramic; CP, ceramic-on-polyethylene; MP, metal-on-polyethylene

The benchmark prosthesis construct at 7 years was identified as the ExeterV40/Trilogy (Stryker Ltd., Michigan, USA) metal on polyethylene. There were 1433 remaining at risk and the failure rate was 2·74% (95% CI 2.08–3.40). There were 25 other constructs with > 500 at risk. Five constructs were identified as inferior to the reference by > 20% relative risk of which 1 was inferior by > 100% relative risk. Fifteen constructs were noninferior to the 100% margin, of which one construct was also noninferior at the 20% margin (Fig. 5).

**Fig. 6:**
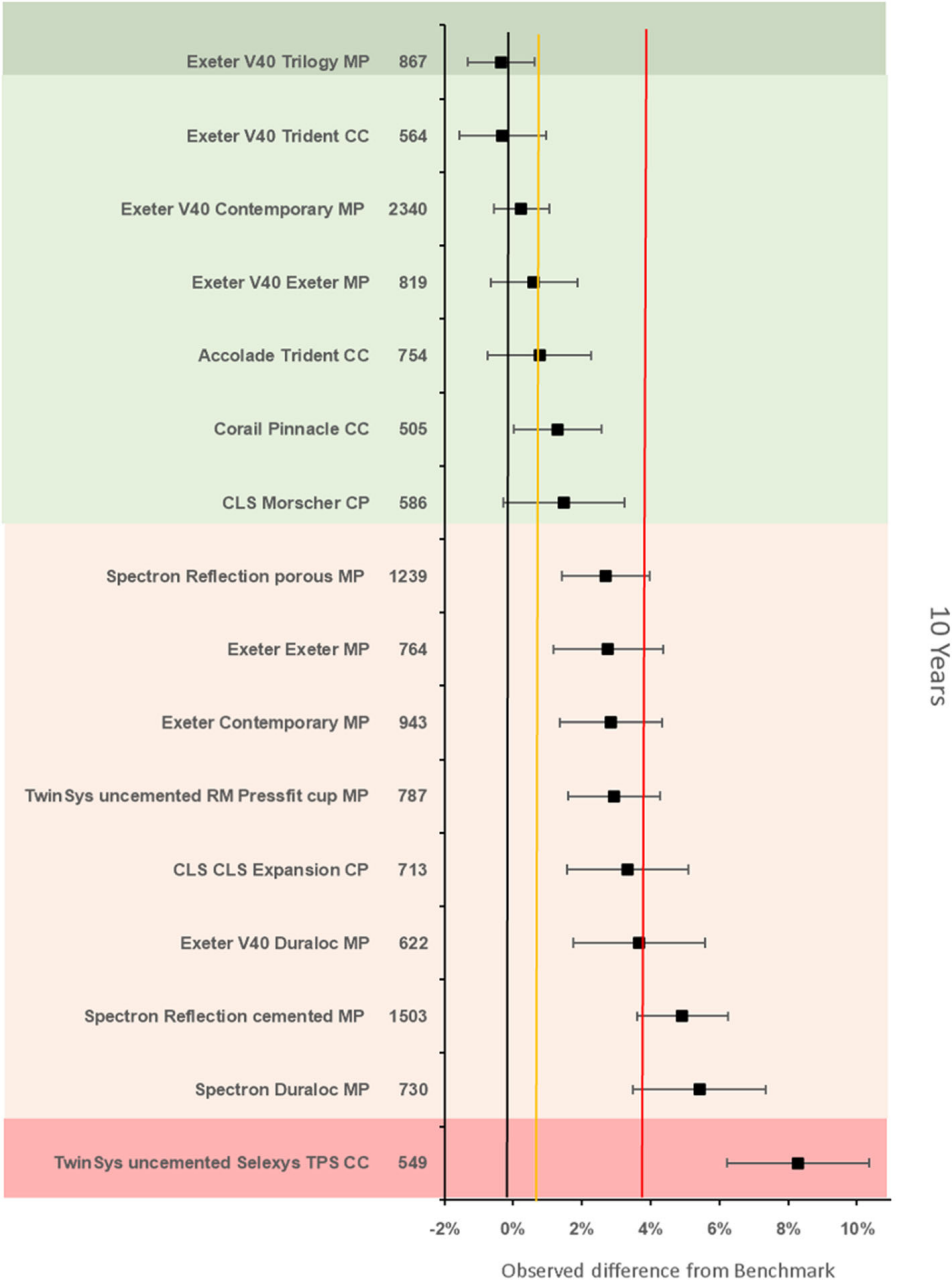
Difference in failure of implanted constructs compared with a contemporary reference (**ExeterV40/Trident MP** (3.79, 95% CI 3.24–4.34)) at 10 years, using all stem-cup combinations with > 500 procedures remaining at risk. CC, ceramic-on-ceramic; CP, ceramic-on-polyethylene; MP, metal-on-polyethylene

The benchmark prosthesis construct at 10 years was identified as the ExeterV40/Trident metal on polyethylene. There were 2082 remaining at risk and the failure rate was 3·79% (95% CI 3·24–4·34). There were 16 other constructs with > 500 at risk. Nine constructs were identified as inferior to the reference by > 20% relative risk of which 1 construct was inferior by > 100% relative risk. Seven constructs were noninferior to the 100% margin, of which one construct was also noninferior at the 20% margin (Fig. 6).

### Noninferiority: analysis by patient gender

There were fewer constructs available for comparison and inferior to benchmark when we performed analysis by gender. In females the reference construct at 3 years was fully cemented (Spectron/Reflection cemented metal on polyethylene (1·00, 95% CI 0·54–1·46) and interestingly the construct inferior at the 100% margin was the Exeter V40/Continuum TM metal on polyethylene. At subsequent time points the reference constructs were the MS 30/Fitmore metal on polyethylene (1·16, 95% CI 0·64–1·67) at 5 years, the Exeter V40/Contemporary metal on polyethylene (2·76, 95% CI 2·22–3·30)) at 7 years and the ExeterV40/Trident metal on polyethylene (3·76, 95% CI 3·02–4·50) at 10 years. No constructs were inferior at the 100% margin at 5, 7 and 10 years compared to the references.

In males the reference construct at 3 years was uncemented (Corail/Pinnacle ceramic on polyethylene (2·04, 95% CI 1·53–2·55)) and at 5, 7 and 10 years the reference construct was the ExeterV40/Trident metal on polyethylene. No constructs were inferior at the 100% margin at 3, 5, 7 and 10 years compared to the references.

At 10 years there were 5 constructs with > 500 constructs at risk in females, 2 were inferior at the 20% margin and 3 were noninferior. In males there were only 2 constructs to compare with the benchmark; 1 was inferior by > 20% and 1 was noninferior (Supplementary Figures).

## Discussion

We have shown in 62,251 primary THRs the relative performance of implanted constructs compared to a contemporary best practice benchmark. There is substantial variation in the performance of THR constructs. A noninferiority approach conveys distinct advantages as opposed to component/years in the NZJR annual reports, standard Kaplan-Meier analyses in the NZJR reports, or categorical grades provided by organisations such as ODEP. There was also, as found by Deere et al., 2019; heterogeneity in constructs used in females and males. Whilst hybrid constructs were benchmarks in both genders there was a trend towards cemented fixation in females and uncemented fixation in males. We were unable to stratify by both gender and age given insufficient numbers at risk.

Our study also shows that the best practice benchmarks were predominantly hybrid constructs with a dual taper polished cemented stem and metal-on-polyethylene bearing and is in accordance with the findings of Gwynne-Jones et al. [15]. In the overall comparisons, the MS30 paired with the Fitmore cup metal on polyethylene was the reference construct at both 3 and 5 years. The Exeter V40 cemented stem paired with the Trilogy (7 years) and Trident (10 years) was the benchmark construct. This study also showed that the Exeter V40/Trident metal on polyethylene was either noninferior or the benchmark at 7 and 10 years whilst the Exeter V40/Trident ceramic on ceramic was noninferior at 3 and 5 years. This strongly suggests that a hybrid construct such as the Exeter V40/Trident combination or a construct noninferior to it could appropriately be used as default options for the majority of patients and this finding is consistent with study by Evans et al., 2020 [16]. This is particularly relevant for inexperienced surgical teams, as they can focus training on, and become expert with, a single prosthesis construct [16]. Potentially this may reduce the risk of technical error, to be cost saving through bulk purchasing arrangements and via a reduction in failure rates. The absolute level of failure of commonly used constructs is relatively low, and < 5% in many instances. Interestingly uncemented constructs compared favorably to hybrid constructs. The Corail/Pinnacle ceramic on ceramic, Accolade/Trident ceramic on ceramic and CLS/Morscher ceramic on polyethylene were noninferior at 10 years compared to the Exeter V40/Trident metal on polyethylene benchmark.

Whilst the Exeter V40 cemented stem had outstanding performance in the NJR study by Deere et al. [5], in our study when paired with the Tritanium cup metal on polyethylene at 3 years and Continuum cup metal on polyethylene at 5 years there was twice the revision rate compared to the reference. There was therefore great variation in construct performance with the Exeter V40 depending on which cup it was used with. This illustrates the need to benchmark constructs as opposed to individual implants which make up prosthesis constructs, which has the potential to provide false reassurance in terms of efficacy as the individual elements of a construct are not independent.

This study has a number of strengths. Firstly, we illustrate the need to compare implant constructs as opposed to individual implant components. Secondly, the unambiguous presentation of data allows surgeons, patients and policy makers to directly compare commonly used prosthesis constructs to a reference construct. The constant application of benchmarking methodology and observed trends across both the New Zealand and English and Welsh National Joint Replacement Registers suggests results are generalizable and will be useful to both patients, surgeons, and policy makers.

Our study has a number of limitations; case-mix adjustment by stratification is difficult to account for. Despite efforts to restrict confounding factors, residual confounding factors may be present. The ability to interpret analyses from a causal perspective is limited. It is also known that revision rate is influenced by factors such as the primary indication and the severity of preoperative hip disease.

The results from this study have implications for the way both practicing surgeons, purchasers and patients approach total hip replacement. The transparent presentation of data performance may be useful for all parties when funding a hip replacement, deciding to have surgery with a particular construct and the likely chances of experiencing a revision. With specific reference to New Zealand policy of subsidization of prosthesis constructs used in public hospitals, it is essential that purchasers have access to all local and globally relevant and independent sources of data to ensure public money is used in the most cost-effective manner possible, ensuring as many patients will be treated as possible within the available budget constraints.

## Conclusions

For new surgeons, or surgeons looking to optimize the care of their patients, they now have an independent and detailed source of data which compares a wide variety of prosthesis constructs using clinically relevant strata. This will ensure they can pick prostheses that match their surgical competencies or reflect on their need to seek further training, for example in the use of particular prostheses, to ensure they can use implants with a strong track record of performance. Lastly, we hope detailed data will be made available to patients in order to facilitate the shared decision-making process required to inform patients of the risk of revision before deciding to undergo surgery.

## Supplementary Information

**Additional file 1.**

## Data Availability

The datasets generated and/or analysed during the current study are available in the New Zealand Joint Register (nzoa.org.nz).

## Abbreviations

NZJR: New Zealand Joint Registry
THR: Total Hip Replacement
PTIR: Prosthesis Time Incidence Rate
ODEP: Orthopaedic Data Evaluation Panel
MP: Metal on polyethylene
CP: Ceramic on polyethylene
CC: Ceramic on ceramic
CI: Confidence Interval
SE: Standard error
