# Supplementary material for: Benchmarking total hip replacement constructs using noninferiority analysis: the New Zealand joint registry study
Source: BMC Musculoskelet Disord. 2021 Aug 21;22:719. doi: 10.1186/s12891-021-04602-0 (PMC8380353; doi:10.1186/s12891-021-04602-0)
Supplement: Supplementary file 1 — Additional file 1. [file 12891_2021_4602_MOESM1_ESM.docx]

**Supplementary Figures:**

**Females:**


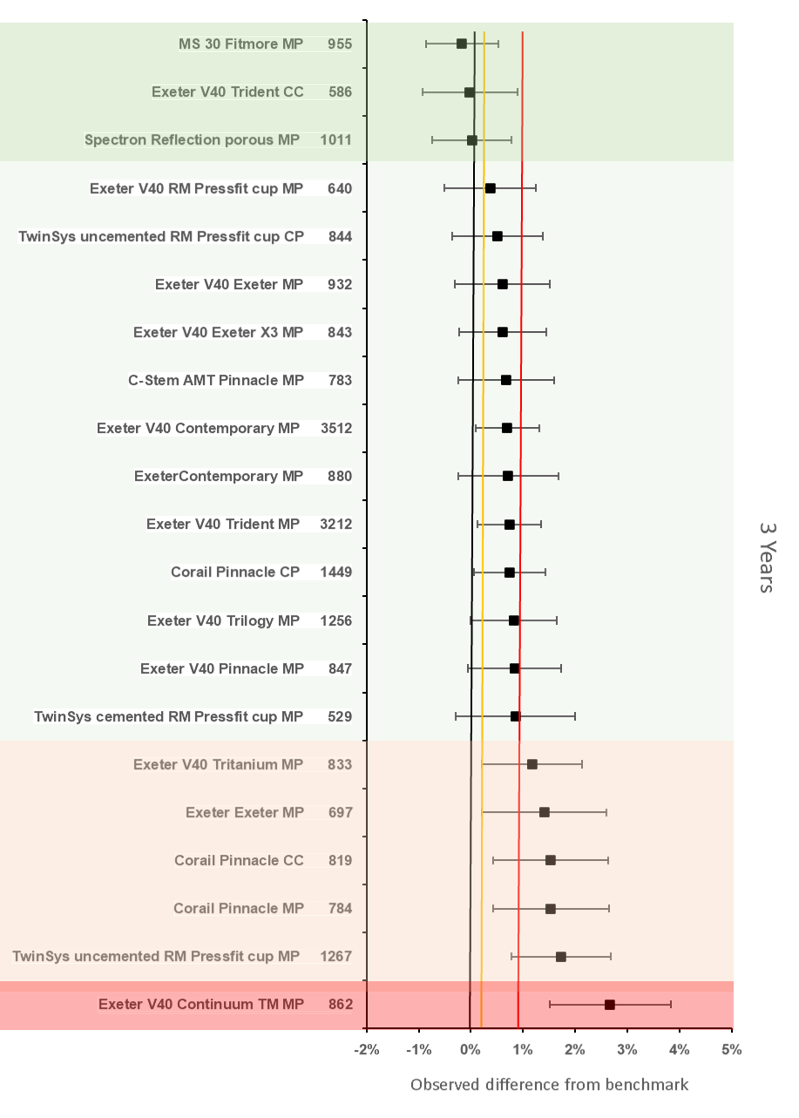


Figure 7. Difference in failure of implanted constructs compared with a contemporary reference (**Spectron Reflection cemented MP** (1^.^00%, 95% CI 0^.^54 - 1^.^46) at 3 years, using all stem-cup combinations with >500 procedures remaining at risk. CC, ceramic-on-ceramic; CP, ceramic-on-polyethylene; MP, metal-on-polyethylene.


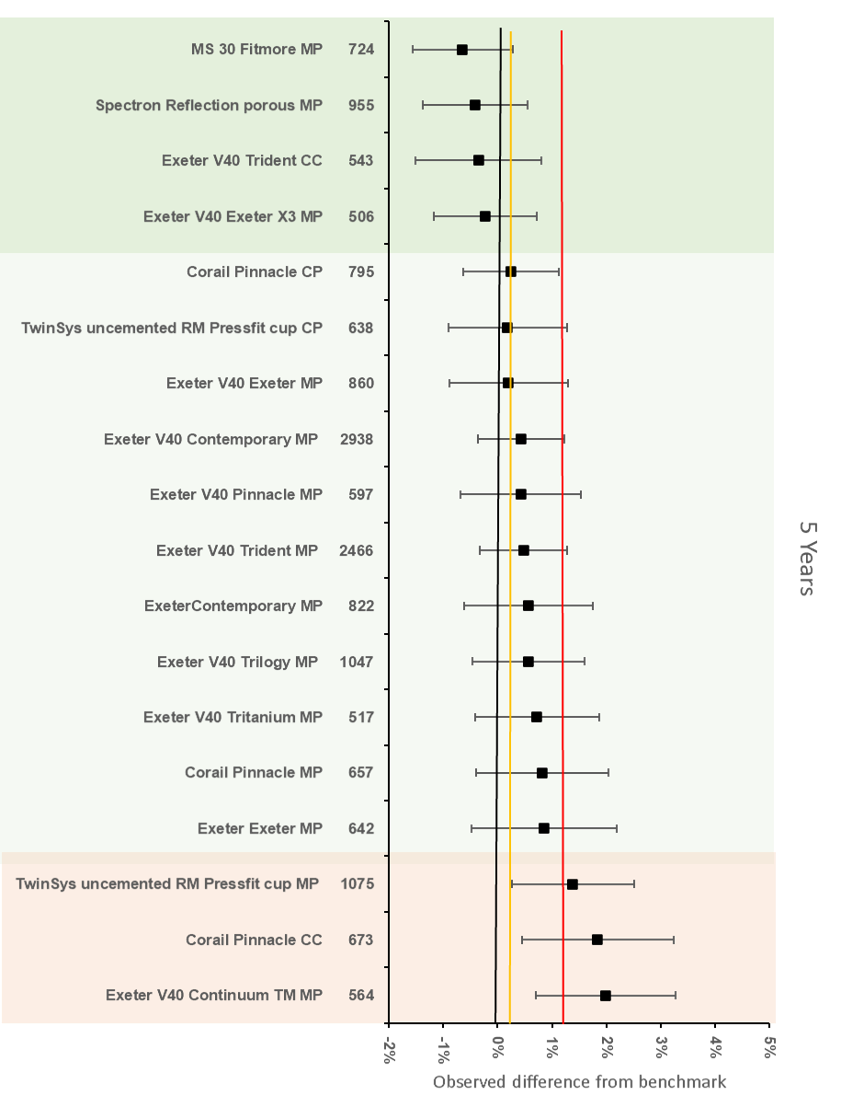


Figure 8 Difference in failure of implanted constructs compared with a contemporary reference (**MS 30 Fitmore MP** (1^.^16%, 95% CI 0^.^64 - 1^.^67) at 5 years, using all stem-cup combinations with >500 procedures remaining at risk. CC, ceramic-on-ceramic; CP, ceramic-on-polyethylene; MP, metal-on-polyethylene.


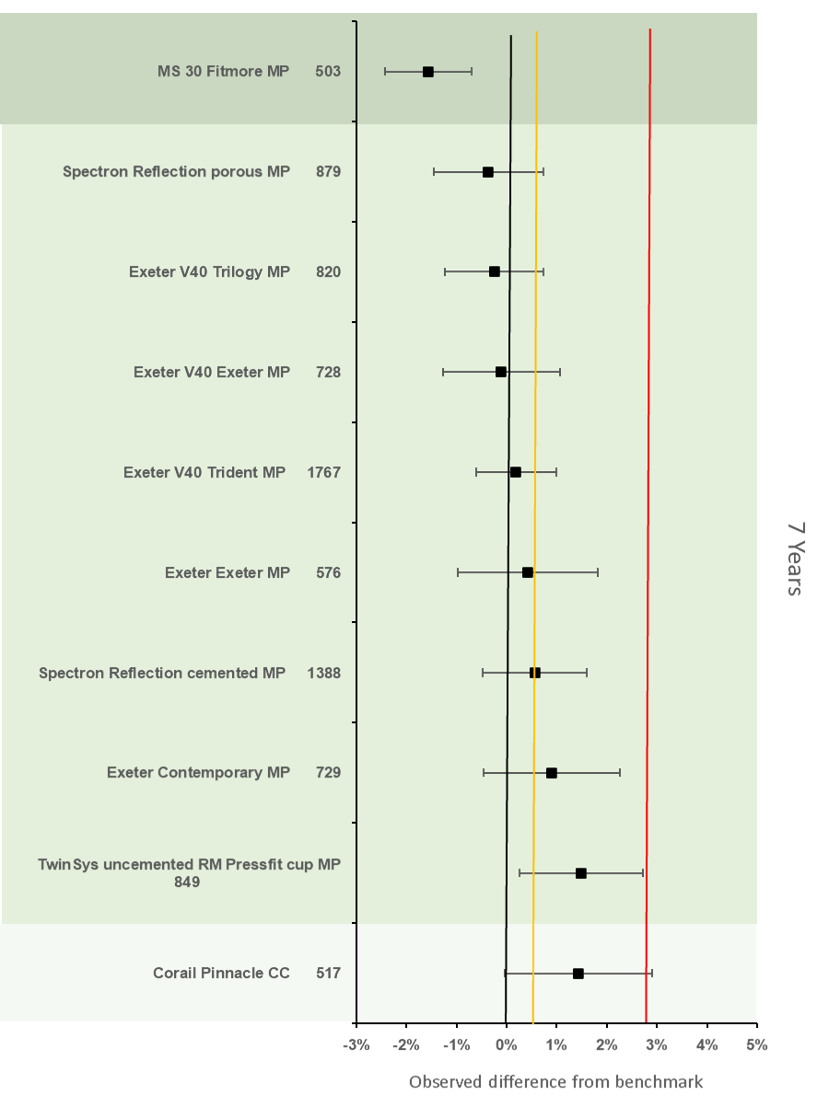


Figure 9. Difference in failure of implanted constructs compared with a contemporary reference (**Exeter V40 Contemporary MP** (2^.^76%, 95% CI 2^.^22 - 3^.^30)) at 7 years, using all stem-cup combinations with >500 procedures remaining at risk. CC, ceramic-on-ceramic; CP, ceramic-on-polyethylene; MP, metal-on-polyethylene.


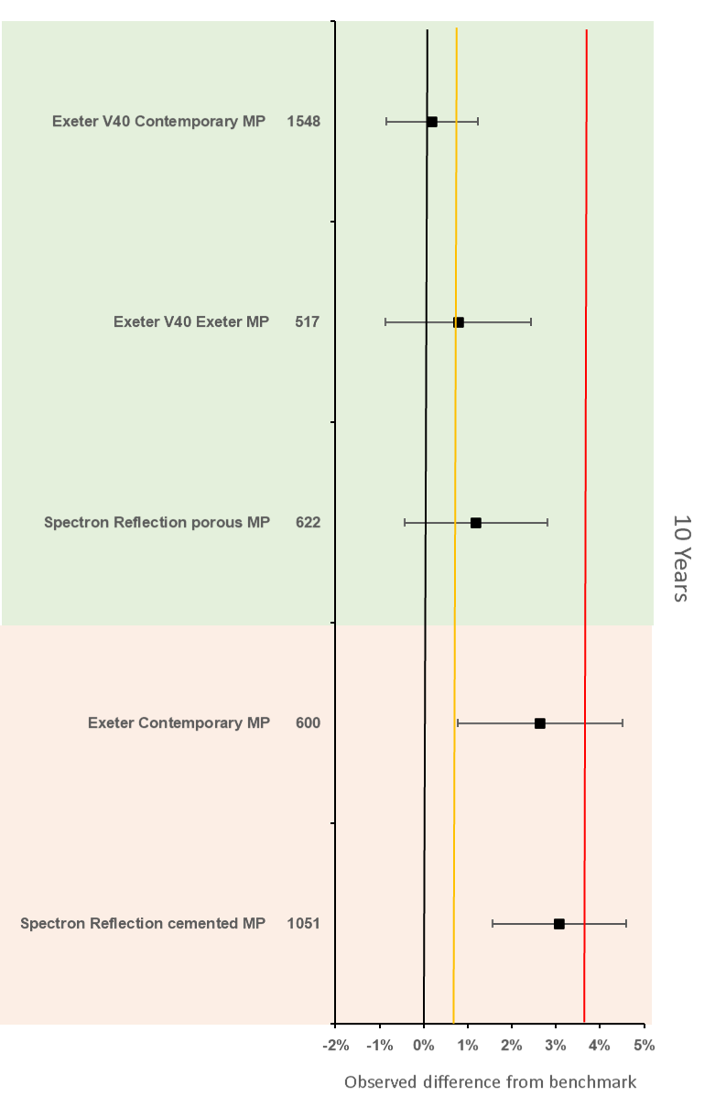


Figure 10. Difference in failure of implanted constructs compared with a contemporary reference (**ExeterV40/Trident MP** (3^.^76%, 95% CI 3^.^02 - 4^.^50)) at 10 years, using all stem-cup combinations with >500 procedures remaining at risk. CC, ceramic-on-ceramic; CP, ceramic-on-polyethylene; MP, metal-on-polyethylene.

**Males:**

**
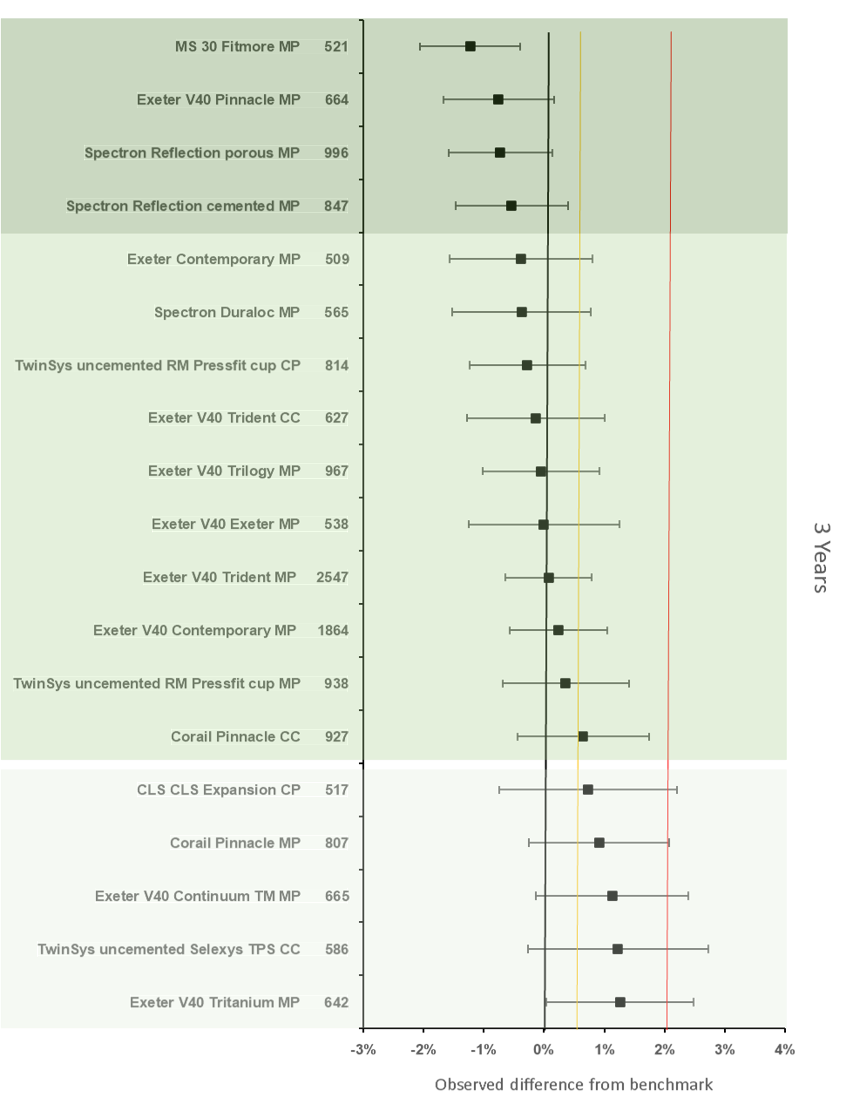
**

Figure 11. Difference in failure of implanted constructs compared with a contemporary reference (**Corail Pinnacle CP** (2^.^04%, 95% CI 1^.^53 - 2^.^55)) at 3 years, using all stem-cup combinations with >500 procedures remaining at risk. CC, ceramic-on-ceramic; CP, ceramic-on-polyethylene; MP, metal-on-polyethylene.


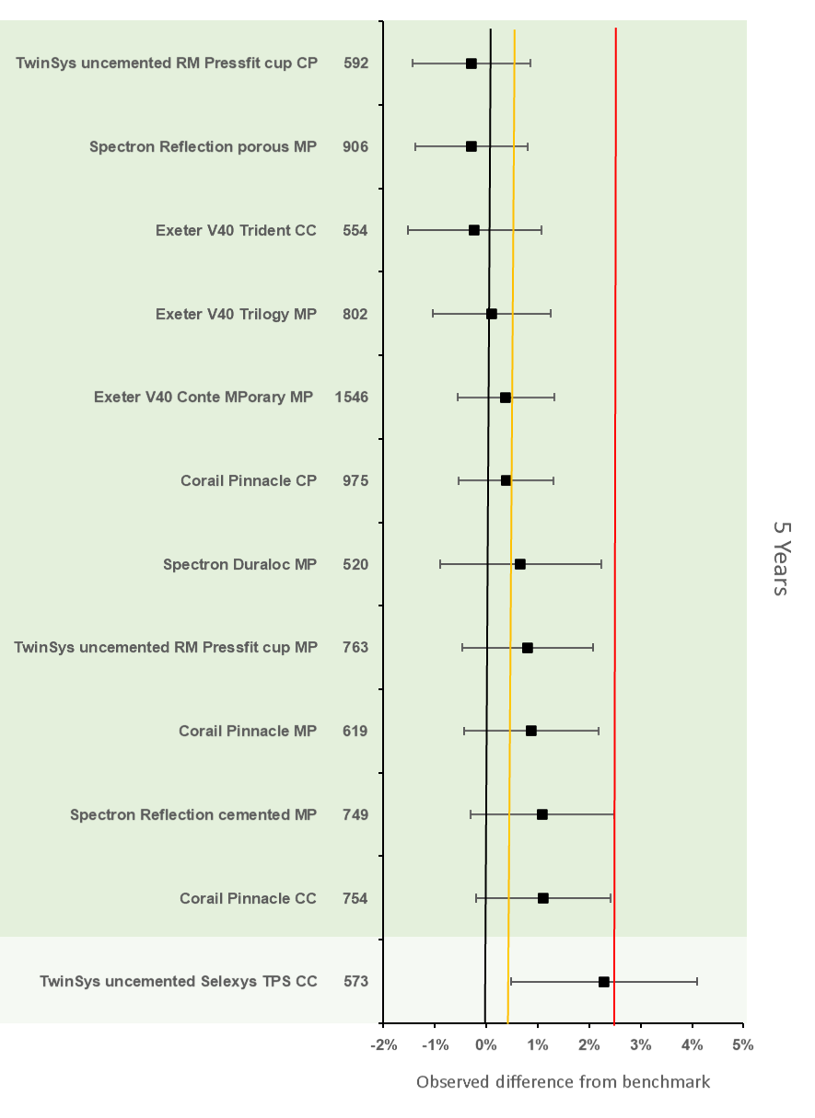


Figure 12. Difference in failure of implanted constructs compared with a contemporary reference (**ExeterV40/Trident MP** (2^.^64%, 95% CI 2^.^06 - 3^.^21)) at 5 years, using all stem-cup combinations with >500 procedures remaining at risk. CC, ceramic-on-ceramic; CP, ceramic-on-polyethylene; MP, metal-on-polyethylene.


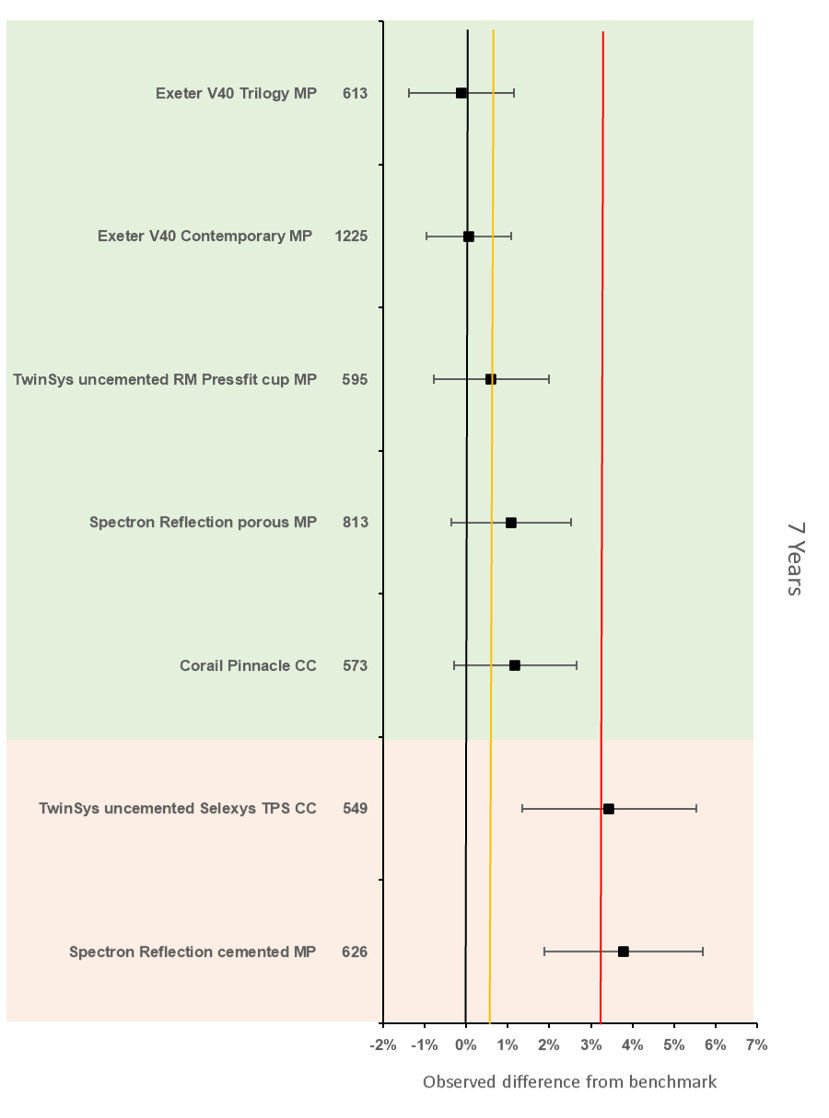


Figure 13. Difference in failure of implanted constructs compared with a contemporary reference (**Exeter V40 Trident MP** (3^.^16%, 95% CI 2^.^49 - 3^.^82)) at 7 years, using all stem-cup combinations with >500 procedures remaining at risk. CC, ceramic-on-ceramic; CP, ceramic-on-polyethylene; MP, metal-on-polyethylene.


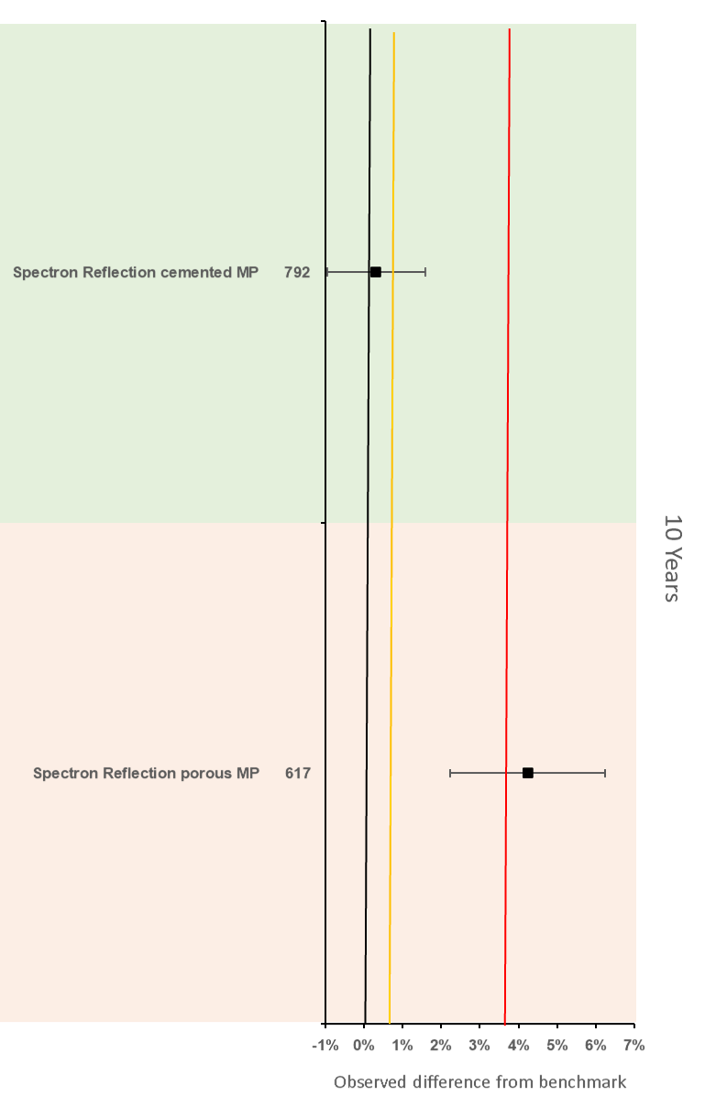


Figure 14. Difference in failure of implanted constructs compared with a contemporary reference (**ExeterV40/Trident MP** (3^.^84%, 95% CI 3^.^03 - 4^.^66)) at 10 years, using all stem-cup combinations with >500 procedures remaining at risk. CC, ceramic-on-ceramic; CP, ceramic-on-polyethylene; MP, metal-on-polyethylene.
